# Coordination of leaf hydraulic, anatomical, and economical traits in tomato seedlings acclimation to long-term drought

**DOI:** 10.1186/s12870-021-03304-y

**Published:** 2021-11-15

**Authors:** Shuang Li, Abdoul Kader Mounkaila Hamani, Yingying Zhang, Yueping Liang, Yang Gao, Aiwang Duan

**Affiliations:** 1 Farmland Irrigation Research Institute, Key Laboratory of Crop Water Use and Regulation, Chinese Academy of Agriculture Sciences, Ministry of Agriculture and Rural Affairs, Xinxiang, Henan 453002 China; 2 Graduate School of Chinese Academy of Agricultural Sciences, Beijing, 100081 China

**Keywords:** Hydraulic traits, Anatomical traits, Economics traits, Stomatal conductance, Mesophyll conductance, Drought

## Abstract

**Background:**

Leaf hydraulic and economics traits are critical for balancing plant water and CO_2_ exchange, and their relationship has been widely studied. Leaf anatomical traits determine the efficiency of CO_2_ diffusion within mesophyll structure. However, it remains unclear whether leaf anatomical traits are associated with leaf hydraulic and economics traits acclimation to long-term drought.

**Results:**

To address this knowledge gap, eight hydraulic traits, including stomatal and venation structures, four economics traits, including leaf dry mass per area (LMA) and the ratio between palisade and spongy mesophyll thickness (PT/ST), and four anatomical traits related to CO_2_ diffusion were measured in tomato seedlings under the long-term drought conditions. Redundancy analysis indicated that the long-term drought decreased stomatal conductance (g_s_) mainly due to a synchronized reduction in hydraulic structure such as leaf hydraulic conductance (K_leaf_) and major vein width. Simultaneously, stomatal aperture on the adaxial surface and minor vein density (VD_minor_) also contributed a lot to this reduction. The decreases in mesophyll thickness (T_mes_) and chlorophyll surface area exposed to leaf intercellular air spaces (S_c_/S) were primarily responsible for the decline of mesophyll conductance (g_m_) thereby affecting photosynthesis. Drought increased leaf density (LD) thus limited CO_2_ diffusion. In addition, LMA may not be important in regulating g_m_ in tomato under drought. Principal component analysis revealed that main anatomical traits such as T_mes_ and S_c_/S were positively correlated to K_leaf_, VD_minor_ and leaf thickness (LT), while negatively associated with PT/ST.

**Conclusions:**

These findings indicated that leaf anatomy plays an important role in maintaining the balance between water supply and CO_2_ diffusion responses to drought. There was a strong coordination between leaf hydraulic, anatomical, and economical traits in tomato seedlings acclimation to long-term drought.

**Supplementary Information:**

The online version contains supplementary material available at 10.1186/s12870-021-03304-y.

## Background

Drought, especially the prolonged and intense one, is one of the leading environmental factors limiting crop productivity and yield worldwide. Drought could seriously restrict agricultural and economic development [1, 2]. Leaves are directly involved in carbon assimilation, respiration, and water relations [3, 4]. Understanding the mechanisms of leaf functional traits can considerably advance our knowledge regarding plant adaptive survival strategies to suit the surrounding conditions. Leaf morphological and physiological plasticity is essential for plants to efficiently utilize finite environmental resources (e.g., soil water, nutrients, and light) when suffering from abiotic stresses [5]. Despite the varieties of leaf traits, those related to water and CO_2_ exchange have gained most of the attention recently [6, 7]. One set of traits form a group of leaf hydraulic traits, such as stomatal traits and venation traits, which indicate how plants balance water demands and supplies under environmental stresses [3, 8, 9]. Another set of leaf traits is strongly associated with the balance between the investments and returns for water or nutrient resources and carbon, such as leaf maximum photosynthetic capacity (A_max_), leaf dry mass per area (LMA) and leaf nitrogen concentration, which are known as the leaf economic characteristics forming the so-called worldwide leaf economic spectrum [4, 10]. Leaf hydraulic and economics traits play a crucial role in influencing material and energy exchange for plant adaptation to climate changes.

Water transport and CO_2_ uptake are two critical physiological processes involving in leaf function and thus influence photosynthetic capacity [11, 12]. Stomata are “gatekeepers’’ on the epidermis of leaves that responsible for the exchange of gases (e.g., water vapor and CO_2_) between plant tissues and the atmosphere [13, 14]. Thus, stomata plays significant roles in the regulation of water and carbon cycling [14, 15]. Among stomatal conductance (g_s_), the stomatal morphology such as stomatal density (SD), stomatal size (SS) and stomatal aperture (SA) contributed to a lot in regulating stomatal behavior response to changing environmental conditions, particularly long-term drought. The long-term drought generally results in smaller stomata and higher density across species [16, 17], which may allow plants to make faster and more rapid responses to minimize the water loss and enhance fine regulation of plant water use. This balance is generally achieved by regulating leaf vein density, which has a critical role in water supply and evaporative demand [3, 8, 9]. Analysis of global variations in leaf functional traits—the leaf economics spectrum—has established that the variation in LMA is strongly associated with plant photosynthetic capacity. It has been documented that LMA would increase, decrease or do not change in response to drought, which exhibits high plasticity between species due to drought alters the pattern of combination between bulk leaf thickness (LT) and leaf density (LD) at the primary level [18, 19]. Though a decoupled relationship between leaf economics and hydraulic traits in rain forest trees was reported by several researchers [20, 21], evidences are now mounting that these two suites are coupled across woody and herbaceous species in response to different water conditions and other environmental factors from fine to global scales [6, 10, 22, 23]. However, the analysis of water and CO_2_ diffusion at the leaf morphological structure level might be too coarse, since those move out or into mesophyll tissues should pass through a series of ultrastructural characteristics, which can directly reflect mechanistic responses related to gas exchange efficiency.

These ultrastructural characteristics form mesophyll anatomical traits, which are often found to be related to the mesophyll conductance to CO_2_ (g_m_) from the intercellular airspace (C_i_) to chloroplasts, such as mesophyll thickness (T_mes_), cell wall thickness (T_cw_), the volume fraction of intercellular air spaces (f_ias_) and chlorophyll surface area exposed to leaf intercellular air spaces per leaf area (S_c_/S) [24–26]. Previous researches suggested that LMA limits photosynthetic efficiency by affecting g_m_ [27–29]. Some reviews and reports also reported that the g_m_ is largely determined by mesophyll anatomic traits in adaptation to long term stresses such as drought [24, 30], potassium deficiency [31] and high leaf-to-air vapor pressure difference (VPD) [32]. On the other hand, leaf hydraulic conductance (K_leaf_), representing the efficiency of water transport through the petiole to leaf vein system, can mediate the covariation of g_s_ and g_m_ responses to environmental factors through the shared diffusion pathways of water and CO_2_ within mesophyll [11, 33]. Therefore, revealing the correlation between anatomical and hydraulic traits as well as economics traits seemed to be more beneficial for us in understanding the physiological mechanisms of leaf functions acclimation to water limitation. However, it remains unclear whether variations in leaf anatomical traits are associated with leaf hydraulic and economics traits acclimation to long-term drought especially within a cultivated crop species. A detailed investigation is required to fill essential gaps in plant selection and breeding for desired agronomic leaf functional traits.

Tomato (Solanum lycopersicum) is one of the most widely cultivated vegetable plants globally and a model crop in agronomic research. Numerous studies have proven that drought affects tomato yield and fruit quality [34, 35]. Understanding the significance of leaf hydraulic, anatomical, and economical traits to the process of trading water and CO_2_ in tomato would be with a great theoretical and practical meaning. To this end, eight key leaf hydraulic traits, four common economics traits and four anatomic traits which related to CO_2_ diffusion efficiency and photosynthesis were studied in this research (Table 1). All these traits are measured or collected on the last day at the end of the experiment. The main objectives of this study were to (1) investigate how leaf hydraulic, economics and anatomical traits respond to intense long-term drought, and to (2) test whether these three leaf traits are coordinated acclimation to drought in the specific cultivated tomato plants.

**Table 1:** The measured leaf functional traits and their categorization

Group	Traits	Abbr.	Unit
Leaf hydraulic traits	Leaf hydraulic conductance	K_leaf_	mmol m^−2^ s^−1^ MPa^−1^
	Minor venation density	VD_minor_	mm mm^−2^
	Major venation density	VD_major_	mm mm^−2^
	Major vein width		µm
	Stomatal density	SD	mm^−2^
	Stomatal size	SS	µm^2^
	Stomatal aperture	SA	µm
	Maximum stomatal conductance to water vapor	g_wmax_	mol H_2_O m^−2^ s^−1^
Leaf economics traits	Leaf dry mass per area	LMA	g m^−2^
	Leaf thickness	LT	mm
	Leaf density	LD	g cm^−3^
	Ratio between palisade and spongy mesophyll thickness	PT/ST	µm µm^−1^
Leaf anatomic traits	Mesophyll thickness	T_mes_	µm
	Cell wall thickness	T_cw_	µm
	Volume fraction of intercellular air spaces	f_ias_	%
	Chlorophyll surface area exposed to leaf intercellular air spaces per leaf area	S_c_/S	m^2^ m^−2^

## Results

### Variations in leaf gas exchange and functional traits to drought

**Fig. 1:**
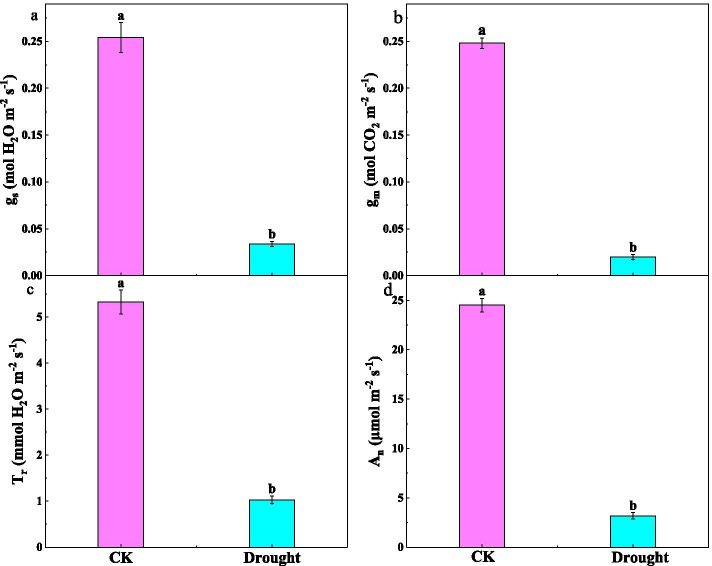
(**a**) Stomatal conductance (g_s_), (**b**) mesophyll conductance (g_m_), (**c**) transpiration rate (T_r_) and (**d**) net assimilation rate (A_n_) for tomato under the well-watered (CK) and drought treatments. Data are means ± standard error (SE) (n=6). Different letters denote statistically significant differences between treatments (*P* < 0.01)

The leaf gas exchange parameters of tomato seedlings were markedly sensitive to drought (Fig. 1). Figure 1a and b illustrated that drought significantly decreased g_s_ and g_m_ by 86.70 % and 92.04 %, respectively. A positive and significant correlation was found between CO_2_ diffusions, T_r_ and A_n_ (Table S1). Consequently, T_r_ and A_n_ were significantly lower under drought than the well-watered treatment (Fig. 1c and d).

**Fig. 2:**
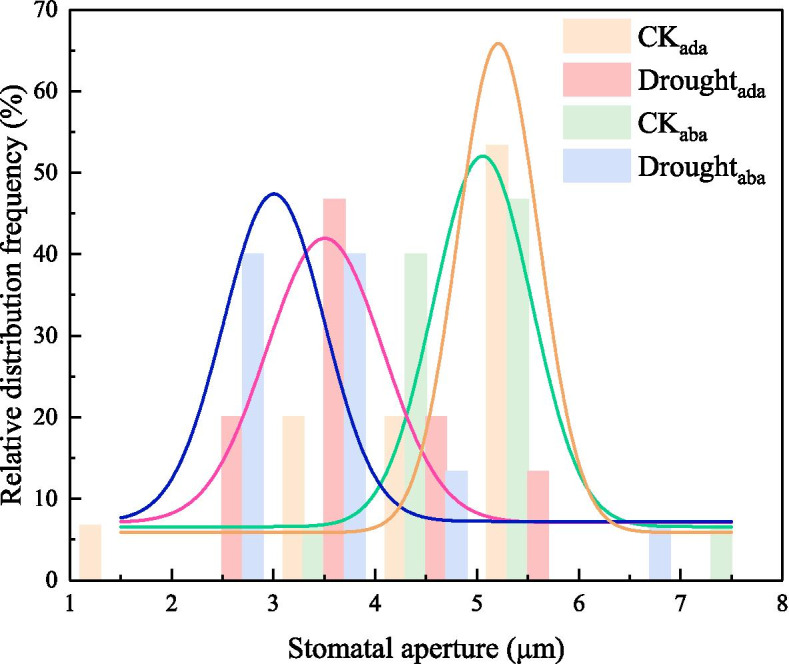
The relative distribution frequency of stomatal aperture on the adaxial and abaxial surface in the well-watered (CK) and drought tomato plants

**Fig. 3:**
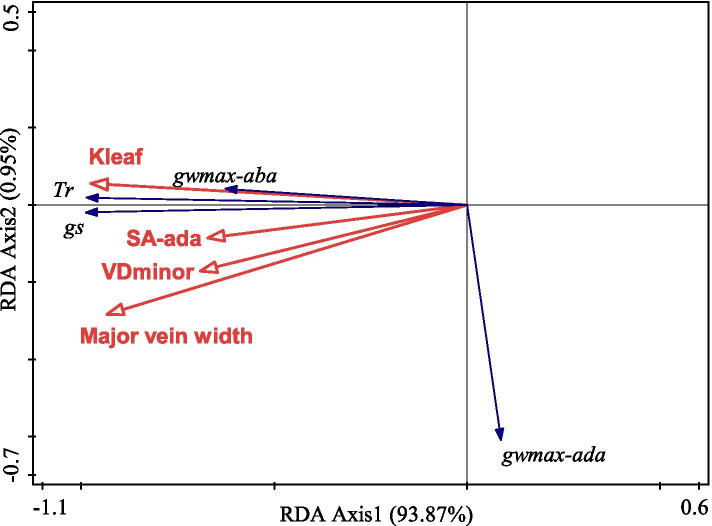
Redundancy analysis (RDA) presenting the association among traits related to water loss, stomatal structural and water supply

Drought reduced the stomatal aperture and size on both the adaxial and abaxial surface of leaves, but no difference of these two traits was observed between the adaxial and abaxial surface in response to drought (Table 2; Fig. S2a-d). The distribution frequency of stomatal aperture fitted a gaussian function (Fig. 2). According to the fitting curve results, the order of stomatal opening size with the highest occurrence frequency was: stomatal aperture on the adaxial surface in CK (CK_ada_) > stomatal aperture on the abaxial surface in CK (CK_aba_) > stomatal aperture on the adaxial surface in drought (Drought_ada_) > stomatal aperture on the abaxial surface in drought (Drought_aba_). From the column results, the relative frequency of 5-5.5 μm aperture in CK_ada_ contributed to 53.33 %. Drought did not affect SD on the abaxial surface, whereas markedly increased SD on the adaxial surface. In contrast, g_wmax_ on the abaxial surface was lower in drought plants compared with CK. Major vein width, VD_minor_ and K_leaf_ were significantly reduced under drought, whereas VD_major_ was unaffected by drought (Table 3). The associations between water loss and stomatal morphology as well as water supply indexes from RDA were presented in Fig. 3. Four key factors affecting the g_s_ and T_r_ were K_leaf_ (*P* = 0.016), major vein width (*P* = 0.004), SA on the adaxial surface (*P* = 0.038) and VD_minor_ (*P* = 0.016), which explained 94.82 % of the total variance.

**Table 2:** Stomatal aperture, stomatal size, stomatal density (SD) and maximum stomatal conductance to water vapor (g_wmax_) on the adaxial and abaxial surfaces of leaves for tomato under the well-watered (CK) and drought conditions

Treatment	stomatal aperture (µm)	stomatal size (µm^2^)	SD (mm^−2^)	g_wmax_ (mol H_2_O m^−2^ s^−1^)
CK_ada_	4.57±0.3 b	50.91±6.85 b	204.18±9.8 c	0.65±0.06 b
CK_aba_	5.30±0.1 a	63.39±3.4 a	291.68±1.7 a	0.98±0.03 a
Drought_ada_	3.73±0.2 c	38.98±1.6 bc	226.56±5.4 b	0.67±0.03 b
Drought_aba_	3.34±0.1 c	32.28±3.1 c	295.08±9.6 a	0.80±0.06 b

**Table 3:** Major vein width, major vein density (VD_major_), minor vein density (VD_minor_) and leaf hydraulic conductance (K_leaf_) for tomato under the well-watered and drought conditions

Treatment	Major vein width (µm)	VD_major_ (mm mm^−2^)	VD_minor_ (mm mm^−2^)	K_leaf_ (mmol m^−2^ s^−1^ MPa^−1^)
CK	289.43±3.9 a	0.043±0.01 a	6.16±0.1 a	5.03±0.2 a
Drought	252.29±1.2 b	0.045±0.003 a	5.59±0.04 b	0.73±0.1 b

**Fig. 4:**
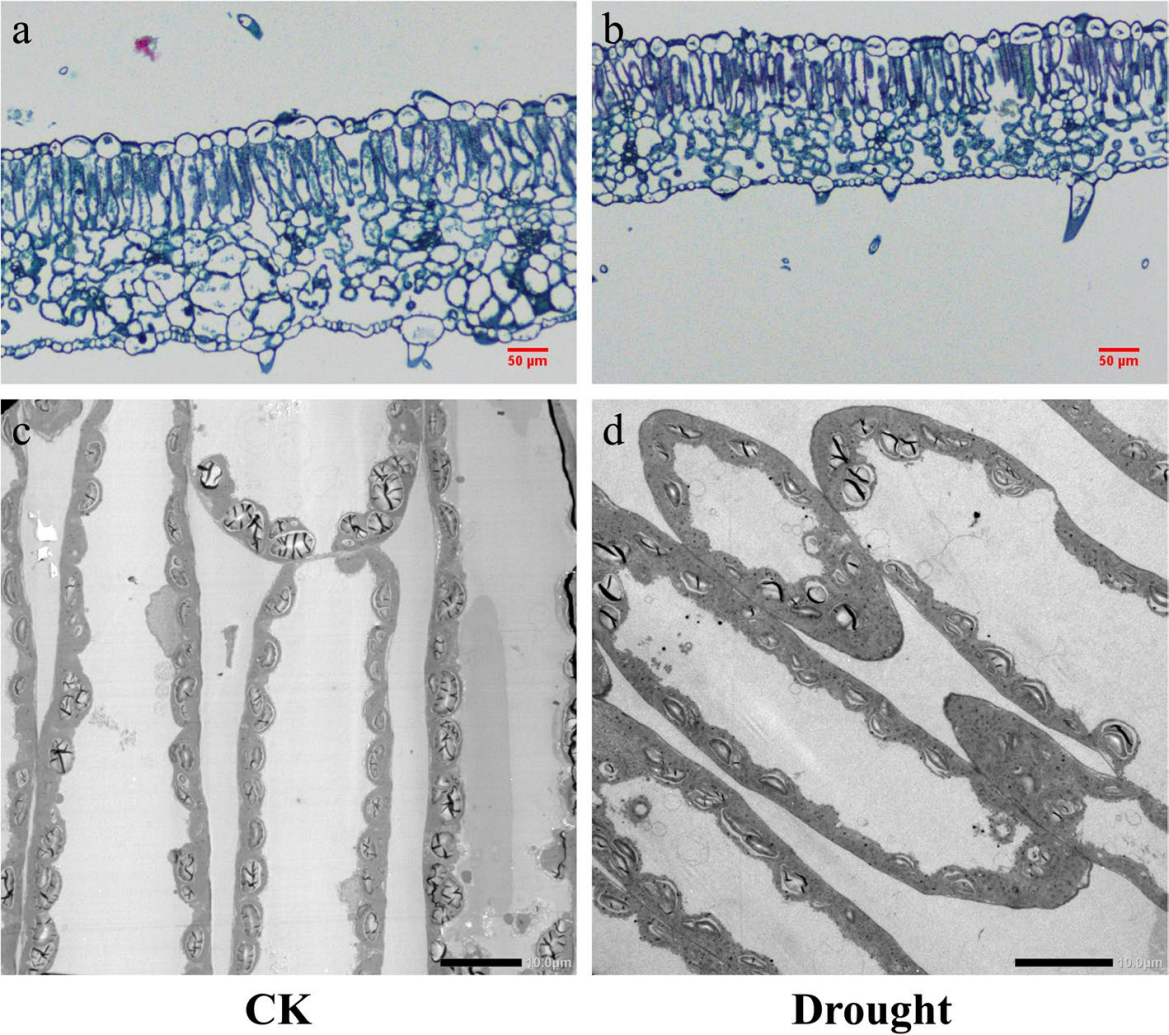
Paraffin vertical sections for leaf (**a**-**b**) and ultrathin section for mesophyll cell structure (**c**-**d**) for tomato seedlings under well-watered (CK) and drought treatments. Scale bars: 1.07 pixels / µm for a-b; 29.7 pixels / µm for c; 35.64 pixels / µm for d

**Fig. 5:**
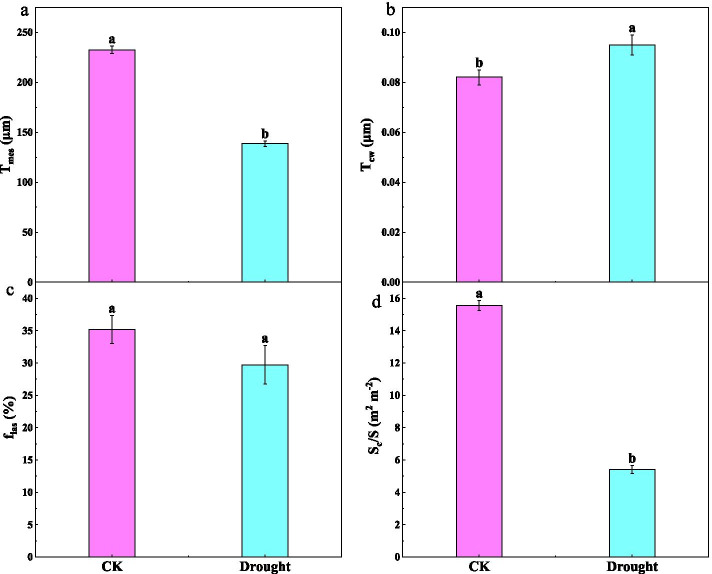
Changes in (**a**) the mesophyll thickness (T_mes_), (**b**) cell wall thickness (T_cw_), (**c**) volume fraction of intercellular air spaces (f_ias_) and (**d**) chlorophyll surface area exposed to leaf intercellular air spaces per leaf area (S_c_/S) in tomato seedlings under the well-watered (CK) and drought treatments. Data are means ± standard error (SE) (n=6). Different letters denote statistically significant differences between treatments (*P* < 0.01)

**Fig. 6:**
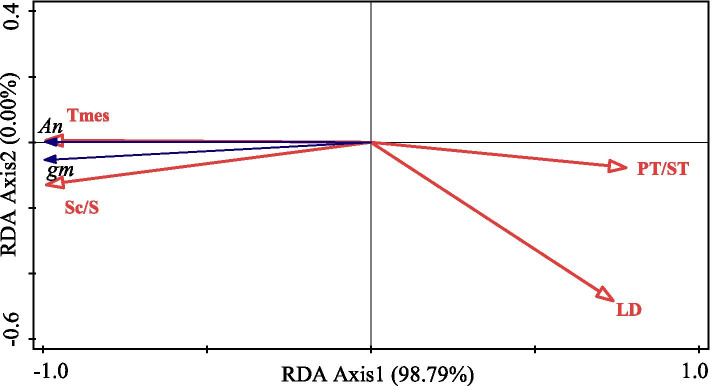
Redundancy analysis (RDA) presenting the association of carbon fixation with leaf economical traits and mesophyll anatomic traits. Trait abbreviations: PT/ST (ratio between palisade and spongy mesophyll thickness), T_mes_ (mesophyll thickness), LD (leaf density), S_c_/S (chlorophyll surface area exposed to leaf intercellular air spaces)

Among the economics traits, the leaf thickness (LT) decreased, while the leaf density (LD) increased in response to drought, whereas LMA was unaffected by drought (Table 4). The ratio between palisade and spongy mesophyll thickness (PT/ST) was higher under drought than CK. Compared to CK, drought significantly decreased T_mes_ (Figs. 4a and b and 5a) and chlorophyll surface area exposed to leaf intercellular air spaces per leaf area (S_c_/S) (Figs. 4c and d and 5d), whereas drought increased T_cw_ (Fig. 5b). There was no significant difference in f_ias_ between in CK and drought treatment (Fig. 5c). The RDA revealed the associations among leaf anatomic, economics traits and carbon fixation (Fig. 6), which accounted for 98.79 % of the variability. T_mes_ and S_c_/S were strongly related to the increase of g_m_ and A_n_. However, PT/ST and LD were negatively correlated with g_m_ and A_n_

**Table 4:** Leaf dry mass per area (LMA), leaf thickness (LT), leaf density (LD) and the ratio between palisade and spongy mesophyll thickness (PT/ST) for tomato seedlings under the well-watered (CK) and drought conditions

Treatment	LMA (g m^−2^)	LT (mm)	LD (g cm^−3^)	PT/ST (µm µm^−1^)
CK	54.37±2.1 a	0.30±0.01 a	0.18±0.01 b	0.65±0.02 b
Drought	50.09±1.5 a	0.25±0.01 b	0.21±0.01 a	0.92±0.07 a

### Coordination among leaf hydraulic, anatomical, and economical traits acclimation to drought

**Fig. 7:**
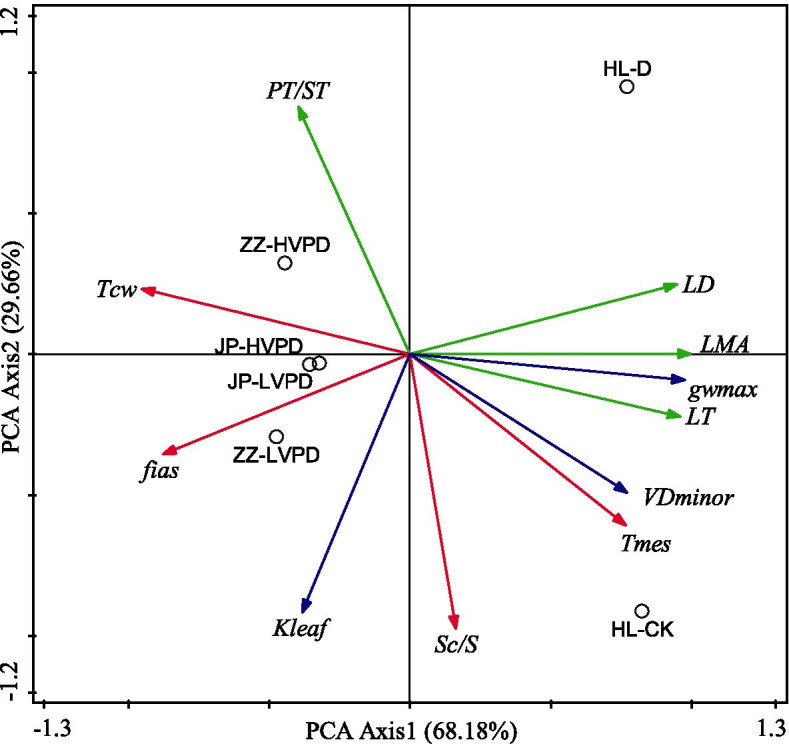
Principal component analyses (PCA) on leaf hydraulic traits (blue lines), anatomical traits (red lines) and economical traits (green lines) by using original data. Values in bracket are percentages explained by the first two components. ZZ and JP refer to Zhongza and Jinpeng tomato cultivars, VPD refers to vapour pressure deficit, HVPD and LVPD refer to high VPD and low VPD. Data of ZZ and JP come from [32]. HL represents Helan tomato cultivars, CK and D mean well-watered and drought-stressed treatments in this study. Data are the means (n=3-6)

To better understand the relationships among leaf hydraulic, anatomical, and economical traits, the principal component analyses (PCA) were performed across three main tomato cultivars in China (Fig. 7). LD, LT, LMA and g_wmax_ loaded in the positive end, while T_cw_ and f_ias_ loaded in the negative end at the first component axis, which accounting for 68.18 % of the total variation. The positive side on the second axis was mostly defined by PT/ST, while the negative side was defined by the K_leaf_, S_c_/S and T_mes_. Together, the first two major axes explained about 97.84 % of the total variation observed in the three tomato cultivars. The results indicated that there was a trade-off among leaf hydraulic traits, economics traits and anatomical traits acclimation to drought across the three main tomato cultivars.

## Discussion

Although drought pretreatment has a priming effect on plant physiological activity [36], our results indicated that A_n_ of tomato seedling under drought was still significantly lower than that under the well-watered conditions (Fig. 1d). Previous studies have suggested that the decline of A_n_ was mainly related to low CO_2_ diffusion conductance [11, 28, 32]. Besides g_s_, g_m_ is also an important factor regulating plant photosynthetic responses to environmental stress, since g_m_ determines the drawdown of CO_2_ from sub-stomatal cavities to chloroplasts [26, 37]. Thus, the reduction in A_n_ was mainly explained by the synchronized decrease in g_s_ and g_m_, which was confirmed by the observed decline in chloroplast CO_2_ concentration (C_c_) (Fig. S1). However, drought unaffected intercellular CO_2_ concentration (C_i_). Although observation of a significant decrease in A_n_ by drought is not new [30, 38], the mechanisms behind the reduction of g_s_ and g_m_ related to anatomic traits induced by drought were systematically and extensively considered in this study.

### Importance of leaf hydraulic traits in determining water loss to long-term drought

Under low water availability, a fundamental challenge for plants will be to balance the transpiration demand and water supply. Stomatal closure acts as an early response to minimize the transpiration water-loss under drought [8, 39]. On the one hand, an efficient water supply within leaves is crucial to maintain stomata opening [7, 8]. According to the RDA model, K_leaf_ was the most important factor affecting g_s_ and T_r_ (Fig. 3). In this case, g_s_ decreased concomitantly accompanied by a reduction in K_leaf_ under drought, in agreement with previous studies [33, 38, 40]. K_leaf_ is composed of two main components, the conductance within the xylem (K_x_) and the conductance through mesophyll tissues outsides the xylem (K_ox_); therefore, an operative xylem conduit is important in facilitating water transport within the xylem [12, 41, 42]. However, during leaf dehydration, air bubbles may invade into vascular system and subsequent blockaded the xylem conduits owing to exceeding the hydraulic safety thresholds caused by the increases in xylem tension under drought [43, 44], which led to a decrease in K_x_. Moreover, although the data of hormonal signals (mainly abscisic acid, ABA) were not shown in the present, it has been proven recently that drought induced ABA accumulation in tomato plants could indirectly increase the risk of hydraulic failure and thus led to induced stomatal closure [45, 46].

On the other hand, leaf vein, as distinct water and nutrients transport systems, plays vital roles in determining the capacity of water transport [3, 6, 47]. In the present study, though VD_major_ was unchanged, the major vein width and VD_minor_ were significantly lower under drought than the well-watered plants, suggesting that drought impeded the vein development and distribution. A small vessel diameter and short vein distribution mean not only a reduction for water transport efficiency from xylem conduits within the bundles sheath cells but also an increased resistance for water movement from the xylem into mesophyll cells [26, 40, 48]. It is not surprising that the coordination among the major vein width, VD_minor_ and K_leaf_ was also noteworthy (Fig. S3 and Fig. 3). Besides the decline of K_x_, lower VD_minor_ tends to decrease K_ox_ due to its effect on hydraulic path length and bundle sheath cell (BS) surface area [49], which deserves more detailed investigation in future work. Hence, leaf xylem anatomy might play a more crucial role in maintaining K_leaf_ and therefore g_s_ acclimation to severe drought.

Last but not least, stomatal morphology including stomatal number and size were suggested to be an adaptive mechanism in plants response to environmental factors such as temperature, water status, VPD, and CO_2_ concentration [9, 15, 17, 50]. As previously reported, leaf stomatal aperture and size on both leaf sides decreased with drought in the present study (Table 2). The smaller stomata tend to open or close more rapidly than larger stomata in response to unpredictable water availability and thus optimize water loss and carbon assimilation [13, 14, 39]. Furthermore, drought increased SD on the adaxial surface that might due to the decrease of stomatal aperture[51]. However, drought did not reduce SD on the abaxial surface as well as g_wmax_ on the adaxial surface. In fact, this higher flexibility of stomatal morphology and distribution may provide an advantage for plants maintenance of photosynthesis under such a severe drought by shortening the pathway for CO_2_ transport from lower leaf surface to the chloroplasts in spongy mesophyll cell [52]. Meanwhile, we also noticed that VD_minor_ and the major vein width were closely coordinated with stomatal aperture on both sides and negatively related to SD in our study (Fig. S3), which was also in accordance with many studies [32, 53]. It is possible that drought restricted the stomatal opening and increased stomatal number to match the balance between in water supply and carbon gain capacity. Consequently, lower water supply reduced g_s_ and T_r_ synchronously.

### Importance of leaf anatomical traits in determining carbon returns to long-term drought

Many authors have reported that leaf anatomical properties play a substantial role in regulating g_m_ [25, 28, 32, 54]. The CO_2_ diffusion efficiency from the intercellular airspaces to chloroplasts is mainly determined by gas and liquid phase conductance. There is evidence that the former is mainly influenced by f_ias_ and T_mes_ [30]. In this study, drought did not affect f_ias_, suggesting that f_ias_ may not exert a significant effect on g_m_. However, the drought-stressed tomato leaves had lower T_mes_ than the well-watered tomato and T_mes_ showed a strong positive correlation to g_m_ (Figs. 5 and 6), mainly due to a thinner mesophyll may decrease the total surface area of CO_2_ assimilation under stress.

Concomitantly, it has been confirmed that liquid phase conductance was primarily responsible for the variation in g_m_, especially for tomato cultivars [25, 31, 32]. In the present study, leaf anatomy of liquid phase such as T_cw_ and S_c_/S were affected by drought. In addition, the strong positive correlation between S_c_/S and g_m_ was observed in Fig. 6 and in other studies [55]. Variability in S_c_/S is closely correlated with the size, amount, and distribution of chloroplasts [24, 56]. Perhaps drought reduced the size, amount, and distribution of chloroplasts, thus decreased the mesophyll surface area. Additionally, a thick cell wall is likely to lengthen CO_2_ diffusion through the plasma lemma [57]. Apart from T_mes_, S_c_/S and T_cw_, other leaf anatomical traits (such as distance from the chloroplasts to the cell wall or chloroplast thickness) and biochemical components in membrane were also affected by drought [24, 26]. Future studies should focus on the comprehensive effects of anatomical traits and biochemical components on drought acclimation.

LMA has been found to be negatively associated with g_m_ in tomato species [29]. In contrast, there was no significant relationship between LMA and g_m_ in this study. The inconsistent results may be due to drought induced an inverse variation between the two components of LMA, i.e., LT and LD. Despite this, the ratio between palisade and spongy mesophyll thickness (PT/ST) was negatively correlated to g_m_ (Fig. 6). Similar trends of change in these variables were also reported by Du et al. [32] in tomato plants, indicating that there was a trade-off between palisade tissues and spongy tissues to acclimate to drought [6], possibly due to drought had a more substantial influence on spongy tissues than palisade tissues.

### Leaf anatomical traits were coordinate with hydraulic and economics traits acclimation to drought

**Fig. 8:**
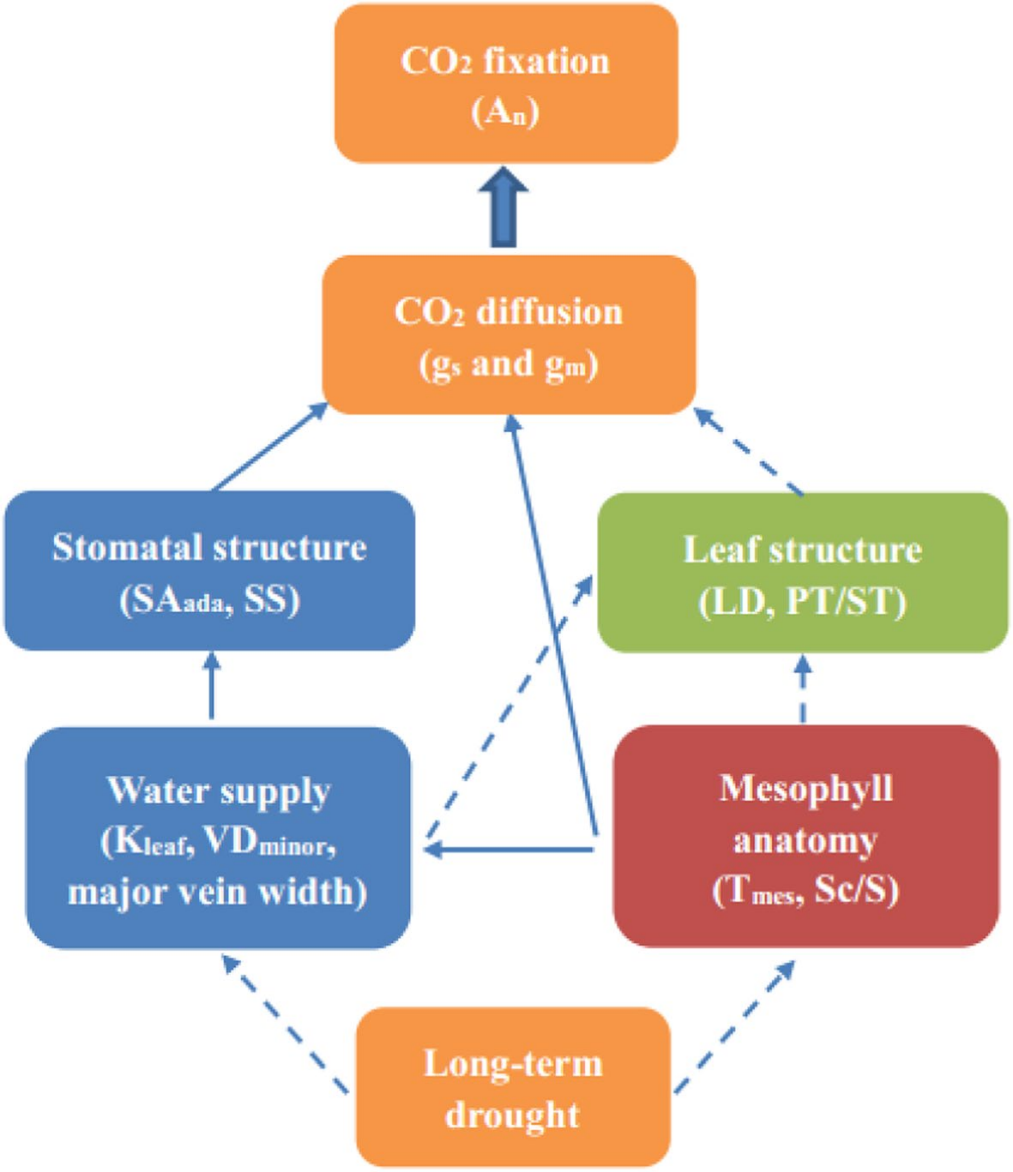
The mechanistic links and coordination between the paired traits in response to long-term drought in this study. Red-shaded variables are related to mesophyll anatomical traits, blue-shaded variables are related to hydraulic trait, green-shaded variables are related to economics trait. Solid arrows indicate positive correlations; dotted arrows indicate negative correlations. All abbreviations are already defined in the text

Yin et al. [6] reported that leaf economics and hydraulic traits were coupled on the Loess Plateau in China, where water availability is a key limiting factor. However, the role of anatomy related to photosynthetic capacity on this coordination is lacking. Thus, we studied the relationships between leaf hydraulic, economics and anatomical traits within three main tomato cultivars (data of another two cultivars from [32] in response to VPD, a measure of the atmospheric demand for water). Similar to soil moisture,VPD has direct influences on stomatal conductance and carbon uptake [58, 59]. According to PCA, mesophyll anatomy (such as T_mes_ and S_c_/S) were positively correlated with hydraulic traits, such as VD_minor_ and K_leaf_ (Fig. 7), these results are in agreement with previous studies [11, 26]. Lower VD and K_leaf_ would make cell loss of turgor, shape and size and thus induce a decline of the photosynthetic surface area during leaf dehydration. This reduction of assimilation capacity that in turn limits the construction of vein, which indicates there is a trade-off between the investment and return of leaf structure. Thus, it is due mainly to the common anatomical structures between non-xylem water transport pathways and CO_2_ diffusion pathways in the mesophyll cell [12]. In addition, these two main mesophyll traits were negatively related to PT/ST, suggesting that a trade-off between leaf anatomicaland economic traits. Relationships of paired traits displayed in Fig. 8 highlight that these anatomical traits play an essential role in coordinating a balance between water supply and CO_2_ uptake acclimation to drought. As the twice drought and re-watering cycles could improve plant tolerance to drought, so more attention should be paid to drought priming effects on leaf hydraulic and mesophyll anatomical changes to long-term drought in future.

## Conclusions

In summary, the results of this study suggested that the decline in g_s_ and T_r_ under the long-term drought condition was mostly responsible for the reduction in stomatal aperture on the adaxial surface primarily due to reduced K_leaf_, major vein width and minor vein density. Drought decreased T_mes_ and S_c_/S, however, increased T_cw_ and PT/ST. Considering g_m_, the decline of T_mes_ and S_c_/S were the crucial causes for g_m_ decrement under drought. Furthermore, these anatomical traits related to hydraulic and economic traits were coordinated in three main tomato cultivars under controlled environment. The present study highlights the important role of leaf anatomy in maintaining the balance between water supply and CO_2_ diffusion in response to drought.

## Materials and methods

### Experimental design

The experimental plants were *Solanum lycopersicum L.*, cv. Helan108, purchased from Jinan Xuechao Seed Co. Ltd, Jinan city in China. The tomato seeds were sown in nursery seedling plate with nursery substrate (sphagnum peat, Pindstrup Mosebrug A/S, Ryomgaard, Denmark). The collection of seeds and the experimental researches on plant were complied with the national guidelines of China. When the second genuine leaf emerged, tomato seedlings were transplanted into 5.3 L pots (height 30 cm, diameter 15 cm) containing 6.5 kg air-dried sandy loam soil. The gravimetric field water capacity (θ_FC_) and wilting point were 22 % (g g^−1^) and 6.8 % (g g^−1^), respectively. To avoid any nutrient deficiency, 1 g N, 0.5 g P and 0.9 g K were applied into each pot. After transplanting, all pots were irrigated to 85 % θ_FC_. Seedlings were cultivated in an environment-controlled chamber with 12 h photoperiod at 600 µmol m^−2^ s^−1^ photosynthetic photon flux density (PPFD). The day/night air temperature was 27–30 °C / 17–19 °C, relative humidity was kept 50-60 %. On the 14th day after transplanting (DAT) with tomato seedlings displaying 3-4 true leaves, water treatments including, well-watered and progressive drought-stressed treatments, were conducted. For the well-watered treatment, soil moisture was maintained around 85 % θ_FC_ during the entire growth period. For the progressive drought-stressed treatment, tomato plants were subjected to progressive soil drying and irrigated to 85 % θ_FC_ when soil moisture decreased to 35 % θ_FC_ aiming to maintain plant survival and growth. At the End of the third drying cycle on the 40th DAT, seedlings have expanded to 8-11 leaves in the flowering stage, all measurements, including gas exchange, hydraulic, economics and anatomical traits were performed on the same new, fully expanded leaves.

### Leaf gas exchange and chlorophyll fluorescence measurements

Leaf gas exchange and chlorophyll fluorescence were measured simultaneously using the Li-Cor 6400 Photosynthesis system (Li-Cor Inc., Lincoln, NE, USA) equipped with an integrated leaf fluorometer chamber head (Li-Cor 6400-40) from 9:00 am to 2:00 pm. During the measurements, PPFD was kept at 1500 µmol m^−2^ s^−1^, the sample CO_2_ concentration was maintained at 400 µmol mol^−1^ using a CO_2_ cylinder. Due to drought has an effect on leaf temperature [60], so the leaf temperature was not controlled during the measurements. Leaf gas exchange and chlorophyll fluorescence were recorded when net photosynthesis (A_n_) was stabilized under these conditions. For each treatment, 6 plants were included.

The actual photochemical efficiency of photosystem II (Φ_PSII_) was determined by measuring steady-state fluorescence (F_s_) and maximum fluorescence (F^′^_m_) during a light-saturating pulse of ca. 8000 mmol m^−2^ s^−1^:

1 $${\Phi_{PS{\rm I}{\rm I}}}={{F_{m}^{'} - {F_s}} {\left/ {\vphantom {{F_{m}^{'} - {F_s}} {F_{m}^{'}}}} \right.} {F_{m}^{'}}}$$

The electron transport rate (J_f_) was then calculated as:

2 $${J_f}={\Phi_{PS{\rm I}{\rm I}}} \times PPFD \times \alpha \times \beta$$

where PPFD was maintained at 1500 µmol m^−2^ s^−1^ on both the well-watered and water-stressed leaves. α represents the leaf absorptance and β reflects the partitioning of absorbed quanta between photosystems I and II.

To estimate α × β, light response curves were measured in these two treatments. PPFD was adjusted in a series of 200, 150, 100, 50, 20 and 0 µmol m^−2^ s^−1^ at 2 % O_2_ by injecting a N_2_ cylinder. During each step of light change, the minimum and the maximum waiting times were 2 and 5 min, respectively. The value of α × β was determined from the linear slope of the relationship between total photosynthesis rate and (PPFD × Φ_PSII_/4) derived from each light point [61].

The variable J method [62] was used to calculate g_m_, as follows:

3 $$g_m=\frac{A_n}{C_i-\frac{\Gamma^\ast(J_f+8(A+R_d))}{J_f-4(A+R_d)}}$$

where C_i_ represents intercellular CO_2_ concentration (µmol CO_2_ mol^−1^), R_d_ represents the light mitochondrial respiration (µmol CO_2_ m^−2^ s^−1^) and was calculated as 1/2 of the dark respiration (R_n_) [33, 63], R_n_ was measured in the dark environment after the light was turned off for 3 h. Γ^*^ is the chloroplast CO_2_ compensation point in the absence of respiration (µmol CO_2_ mol^−1^) and calculated according to [64] .

### Measurements of leaf hydraulic traits

Leaf hydraulic conductance (K_leaf_) was calculated from the transpiration rate (T_r_) and the water potential gradient between water potential of distilled water (Ψ_water_) and leaf water potential (Ψ_leaf_) [26], Ψ_leaf_ was measured by a WP4C Dew point Potential Meter (Decagon, Pullman, WA, USA).

4 $$K_\text{leaf}=\frac{T_r}{\Psi_{water}-\Psi_{leaf}}$$

In this study, major veins referred to veins that extend from the petiole. According to [53], major vein density (VD_major_) was calculated as the total length of the major veins per leaf area. To measure minor vein density (VD_minor_), 1 cm^2^ pieces were cut from the center of each leaf (avoiding major veins) and placed in 10 % NaOH solution. Samples were placed in a 90-degree water bath for 40 min. Each sample was washed in distilled water and then stained in 1 % safranin for 30–60 s. The samples were then put onto glass slides and photographed under a light microscope Teelen XSP 360 A (Teelen Inc., Shanghai, China) at ×4 magnifications. VD_minor_ (mm mm^−2^) was calculated as the total length of leaf veins per leaf area using Image J software (National Institutes of Health, Bethesda, MD, USA). A piece near to 2 cm from the petiole (including the major veins) was also cut to measure the major vein width. For each treatment, three replications were selected and VD_minor_ was averaged with two different fields of views. Leaf adaxial and abaxial epidermis were removed using forceps and then placed in absolute ethyl alcohol for 10 min, samples were mounted upside down on a glass slide. Stomatal anatomical traits including stomatal aperture and length were observed at ×400 magnification in three randomly selected of views, stomatal density (SD) was observed under a light microscope at ×100 magnification. SD was calculated as the total numbers of stomata per area using Image J software. Anatomical maximum stomatal conductance to water vapor (g_smax_, mol H_2_O m^−2^ s^−1^) were then calculated according to [51] as follows:

5 $$g_{smax}=\frac{d\times SD\times\alpha_{max}}{\nu(L+\frac{\pi}{2}\sqrt{\frac{\alpha_{max}}\pi})}$$

where d is the diffusivity of water in air (24.6 × 10^−6^ m^2^ s^−1^ at 25 °C), ν is the molar volume of the air (24.4 × 10^−3^ m^3^ mol^−1^ at 25 °C and 101.3 kPa), L is the stomatal pore depth, which was approximated as (stomatal length/2) and α_max_ is the maximum area of the open stomatal pore (m^2^), which was calculated as π × (stomatal length/4)^2^.

### Measurements of leaf morphological traits

Leaf area was measured using the LI-3000 C Area Meter (Li-Cor Inc., Lincoln, NE USA). Leaf thickness (LT, mm) was measured as an average of the total thickness of leaves using digital Vernier calipers (SATA Tools Co., Ltd, Shanghai, China), avoiding the influence of leaf major veins. Leaf samples were then oven-dried to a constant weight at 75 °C and their biomass was recorded. Leaf dry mass per area (LMA, g m^−2^) was calculated dry mass per leaf area. Leaf tissue density (LD, g cm^−3^) was calculated as the ratio of LMA to LT. For each treatment, six replications were made.

### Measurements of leaf anatomic traits

Following gas exchange measurements, leaf segments (1 mm^2^) were cut from the central leaflet regions, avoiding the veins, and then fixed with 2.5 % glutaraldehyde (v / v) in 0.1 M phosphate buffer for 4 h and washed three times using 0.1 M phosphate buffer (pH=7) for 15 min. The leaf material was fixed in 1 % osmium tetroxide for 2 h at 4 °C. Then, the samples were dehydrated with a graded ethanol series (50, 70, 80, 90, 95 % and 100 %) embedded in Spurr’s resin. Paraffin (6 μm) for light microscopy and ultrathin (50 nm) for transmission electron microscopy (TEM) sections were cut with a Leica RM2016 ultra microtome (Leica Microsystems, Wetzlar, Germany). The light microscope sections were stained with safranin O and fast green and viewed with magnifications of ×100 and ×200 under a light microscope with a Nikon DS-U3 digital camera (Nikon Incorporation, Tokyo, Japan) to measure palisade thickness, spongy thickness, mesophyll thickness, the total cross-sectional area of mesophyll cells (Σ S_s_) and the total length of mesophyll cell exposed to the intercellular air space (L_mes_). The ultrathin cross-sections were imaged using a transmission electron microscope at magnifications of ×500-800 to measure chloroplast distribution and ×10,000−15,000 to measure cell wall thickness (T_cw_). Two leaves from different plants of each treatment were analyzed. The volume fraction of intercellular air spaces (f_ias_), surface area of mesophyll exposed to intercellular air spaces per leaf area (S_m_/S), and chloroplast surface area exposed to intercellular air space per leaf area (S_c_/S) were calculated according to [54]:

6 $$f_{ias}=1-\frac{{\displaystyle\overset{\mathrm o}{\mathrm a}}\;S_s}{T_{mes}W}$$

7 $${S_{mes}}/S=\frac{{{L_{mes}}}}{W}F$$

8 $${S_c}/S=\frac{{{L_c}}}{{{L_{mes}}}}{S_{mes}}/S$$

where W is the width of the measured section (µm), T_mes_ is the mesophyll thickness between two epidermises (µm), L_c_ is the length of chloroplasts exposed to the intercellular air space (µm), and F is the curvature correction factor being 1.42 according to [54].

#### Statistical analysis

Statistical analyses were performed using SPSS 16.0 (IBM SPSS Statistics, Chicago, IL, USA). Normality and equal variances were tested before analysis of variance. The significance of differences between CK and the drought-stressed treatment were analyzed using the one-way analysis of variance (ANOVA) according to Duncan’s multiple range tests at *P* < 0.05 or *P* < 0.01. Pearson correlation coefficients (*r*) were calculated to examine the relationships between key traits. Further, multivariate associations of leaf traits were analyzed using principal component analysis (PCA) in CANOCO 5 (Microcomputer Power, Ithaca, NY, USA). Redundancy analysis (RDA) was used to assess the relationships among leaf water loss, stomatal structure and water supply as well as among carbon fixation, leaf economic and anatomic traits in CANOCO 5. All graphics were performed in Origin-Pro 2017 (Origin Lab, Northampton, MA, USA).

## Supplementary Information

**Additional file 1.**

## Data Availability

The data that support the findings of this study are available from the corresponding author upon reasonable request.

## Abbreviations

T_r_: transpiration rate
g_s_: stomatal conductance
SD: stomatal density
SS: stomatal size
SA: stomatal aperture
aba: leaf abaxial side
ada: leaf adaxial side
K_leaf_: leaf hydraulic conductance
VD_major_: major vein density
VD_minor_: minor vein density
Ψ_water_: water potential of distilled water
Ψ_leaf_: leaf water potential
g_wmax_: the calculated maximum stomatal conductance to water vapor
PPFD: photosynthetic photon flux density
DAT: days after transplanting
A_n_: net photosynthetic rate
A_max_: leaf maximum photosynthetic capacity
α: leaf absorptance
β: the partitioning of absorbed quanta between photosystems I and II (PSI and PSII)
J_f_: electron transport rate
d: the diffusivity of water in air
ν: the molar volume of air
L: the stomatal pore depth
α_max_: the maximum area of the open stomatal pore
Φ_PSII_: actual photochemical efficiency of photosystem II
F_s_: steady-state fluorescence
F^′^_m_: maximum fluorescence
S_m_/S: surface area of mesophyll exposed to intercellular air spaces per leaf area
ΣS_s_: the total cross-sectional area of mesophyll cells
L_mes_: the total length of mesophyll cell exposed to the intercellular air space
W: the width of the measured section
T_mes_: the mesophyll thickness between two epidermises
L_c_: the length of chloroplasts exposed to the intercellular air space
F: the curvature correction factor
LMA: leaf dry mass per area
LT: leaf thickness
LD: leaf density
PT/ST: ratio between palisade and spongy mesophyll thickness
g_m_: mesophyll conductance to CO_2_
T_cw_: cell wall thickness
f_ias_: the volume fraction of intercellular air spaces
S_c_/S: the chlorophyll surface area exposed to leaf intercellular air spaces per leaf area
VPD: vapor pressure difference
CK: well-watered
