# Supplementary material for: Coordination of leaf hydraulic, anatomical, and economical traits in tomato seedlings acclimation to long-term drought
Source: BMC Plant Biol. 2021 Nov 15;21:536. doi: 10.1186/s12870-021-03304-y (PMC8591842; doi:10.1186/s12870-021-03304-y)
Supplement: Supplementary file 1 — Additional file 1. [file 12870_2021_3304_MOESM1_ESM.docx]

**Supplementary Materials**

**Fig. S1.** Intercellular CO_2_ concentration (C_i_) and chloroplast CO_2_ concentration (C_c_) for tomato under well-watered (CK) and drought. Data are means ± standard error (SE) (n=6). Different letters denote statistically significant differences between treatments (*P* < 0.01).


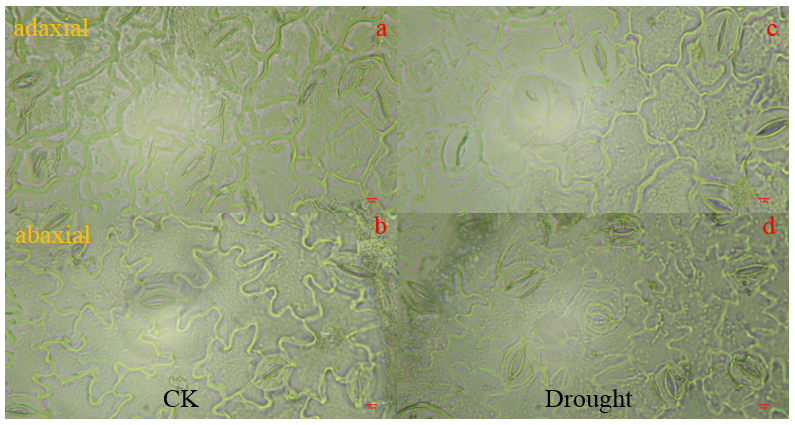


**Fig. S2.** The stomatal structure in the well-watered (CK) and drought tomato plants. Scale bars: 12 pixels / μm for a-d.

**Fig. S3.** Principal component analysis (PCA) of stomatal morphology traits and leaf hydraulic traits. Trait abbreviations: SS (stomatal size), SA (stomatal aperture), aba (leaf abaxial side), ada (leaf adaxial side).

**Table S1** Pearson correlation coefficients among gas exchange parameters.

| Treatment | g_s_ | g_m_ | T_r_ | A_n_ |
| --- | --- | --- | --- | --- |
| g_s_ | 1 | 0.998** | 0.994** | 0.949** |
| g_m_ |  | 1 | 0.964** | 0.998** |
| T_r_ |  |  | 1 | 0.958** |
| A_n_ |  |  |  | 1 |
